# Temporal Susceptibility of Grapevine Pruning Wounds to Botryosphaeriaceae Host-Jumping Pathogens in Central Chile

**DOI:** 10.3390/jof12060424

**Published:** 2026-06-11

**Authors:** Yadira Hernández, Fernanda B. Núñez, Yuramis Quesada, Mauricio Lolas, Karina Elfar, Akif Eskalen, Felipe Gainza-Cortés, Pedro E. Gundel, Eugenio Sanfuentes, Gonzalo A. Díaz

**Affiliations:** 1 Laboratory of Fruit Pathology, Faculty of Agricultural Sciences, University of Talca, Talca 3460000, Chile; yadira.hernandez@utalca.cl (Y.H.); fnunez@utalca.cl (F.B.N.); yuramis.quesada@utalca.cl (Y.Q.); mlolas@utalca.cl (M.L.); 2 Department of Plant Pathology, University of California, Davis, CA 95616, USA; kdelfar@ucdavis.edu (K.E.); aeskalen@ucdavis.edu (A.E.); 3 Viña Concha y Toro S.A., Center for Research and Innovation, Fundo Pocoa s/n, Km10 Ruta K-650, Pencahue 3550000, Chile; felipe.gainza@conchaytoro.cl; 4 Centro de Ecología Integrativa, Instituto de Ciencias Biológicas, University of Talca, Campus Talca, Av. Lircay s/n, Talca 3460000, Chile; pedro.gundel@utalca.cl; 5 Instituto de Biología Funcional y Biotecnología (BIOLAB)-INBIOTEC-CONICET-CICBA, Facultad de Agronomía, Universidad Nacional del Centro de la Provincia de Buenos Aires (UNICEN), Av. República de Italia # 780, Azul 7300, Buenos Aires, Argentina; 6 Facultad de Ciencias Forestales y Centro de Biotecnología, Universidad de Concepción, Edmundo Larenas, 219, Concepción 4070409, Chile; esanfuen@udec.cl

**Keywords:** grapevine pruning wounds, Botryosphaeriaceae, susceptibility period, cross-host infection

## Abstract

Botryosphaeria dieback, caused by species of Botryosphaeriaceae, causes significant economic losses by infecting pruning wounds in vineyards and fruit trees. Previous studies have shown that pruning wounds constitute the main entry point for Botryosphaeriaceae and that isolates from different fruit hosts can infect these tissues regardless of origin. This study assessed the temporal susceptibility of *Vitis vinifera* pruning wounds in four cultivars (Cabernet Sauvignon, Malbec, Merlot, and Sauvignon Blanc) to six Botryosphaeriaceae isolates from different fruit hosts (grapevine, apple, blueberry, and walnut) under greenhouse and field conditions in central Chile. Pruning wounds were inoculated at 1, 15, 30, 45, and 60 d after pruning, and lesion length and wound infection (%) were evaluated. Both variables decreased with increasing wound age in greenhouse and field trials. Wounds were most susceptible during the first 15 d after pruning, with a marked reduction thereafter, although susceptibility persisted up to 60 d. *Neofusicoccum parvum* and *N. arbuti* showed the highest aggressiveness. All isolates were able to infect pruning wounds regardless of host of origin. These results indicate that pruning wounds remain susceptible for an extended period and highlight the importance of considering both wound age and cross-host inoculum sources in disease management strategies.

## 1. Introduction

Grapevine trunk diseases (GTDs) are among the most important threats to vineyard longevity and productivity worldwide [1,2,3,4]. Among them, Botryosphaeria dieback, caused by several species within the family Botryosphaeriaceae, produces internal wood necrosis, shoot dieback, and significant yield losses in many grape-growing regions [5,6,7,8,9,10,11]. In Chile, species such as *Diplodia seriata*, *D. mutila*, *Neofusicoccum australe*, *N. parvum*, and *Spencermartinsia viticola* have been reported from symptomatic grapevines in several viticultural regions [12,13,14,15,16].

Infection by Botryosphaeriaceae species typically occurs through fresh pruning wounds, which act as the main entry points for airborne or splash-dispersed conidia produced in pycnidia on infected wood and pruning debris [1,3,17,18]. Previous studies conducted in different viticultural regions have shown that pruning wounds are highly susceptible to infection immediately after pruning and that susceptibility gradually decreases over time, although it may persist for several weeks depending on environmental conditions [9,17,18,19,20,21,22].

Despite the widespread presence of Botryosphaeriaceae species in Chilean vineyards, the duration of pruning wound susceptibility under the winter conditions of central Chile remains poorly understood. In addition, several Botryosphaeriaceae species occurring on apple, blueberry, and walnut have recently been shown to infect grapevine tissues [23], suggesting a potential cross-host infection risk among neighboring fruit crops in central Chile.

Therefore, this study aimed to determine the temporal susceptibility period of grapevine pruning wounds to infection by Botryosphaeriaceae host-jumping pathogens under greenhouse and field conditions in central Chile.

## 2. Materials and Methods

### 2.1. Fungal Isolates and Inoculum Preparation

Six isolates belonging to the Botryosphaeriaceae family were used in this study, which were obtained from branches or cordons showing canker and dieback symptoms on different fruit tree hosts. The isolates included: *D. seriata* from apple (isolate: Bot_2017_DS3, code: Ds-apple, location: Molina, 35°50′ S, 71°36′ W); *D. seriata* from grapevine (isolate: Bot-2016-DS1-Vid, code: Ds-vine, location: San Clemente, 35°32′ S, 71°29′ W); *N. arbuti* from apple (isolate: Bot-2018-NA32, code: Na-apple, location: Parral, 36°08′ S, 71°49′ W); *N. parvum* from grapevine (isolate: Bot-2016-NP10-Vid, code: Np-vine, location: Curicó, 34°58′ S, 71°15′ W); *Dothiorella sarmentorum* from walnut (isolate: Dsar-2, code: Dsar-wal, location: San Rafael, 35°18′ S, 71°30′ W); and *D. mutila* from blueberry (isolate: Bot-2018-DM16-Ara, code: Dm-blue, location: Linares, 35°50′ S, 71°35′ W). The isolates were identified based on morphological and molecular characteristics [13,24,25,26] and were cultivated and maintained on 2% potato dextrose agar (PDA) (Merck S.A., Darmstadt, Germany) at 20 °C under a 12 h light/dark photoperiod at the Plant Pathology Laboratory, Faculty of Agricultural Sciences, University of Talca, Chile. Mycelial suspensions were prepared by adding 3 mL of 0.1% Tween 80 (Sigma-Aldrich, St. Louis, MO, USA) to Petri dishes containing 7-day-old mycelial cultures. The surface of the media was gently scraped with a sterile scalpel and fragments were ground using a blender (Moulinex LM233A, Alençon, France) for 1 min at speed 5, then the mixture was filtered through two layers of cheesecloth and adjusted to a concentration of 10^5^ mycelial fragments/mL using a hemocytometer (Precicolor, HGB, Gießen, Germany) [27]. Suspensions were prepared 12 h prior to inoculation and stored at 4 °C until use.

### 2.2. Susceptibility of Grapevine Pruning Wounds to Botryosphaeriaceae Infection Under Greenhouse Conditions

Healthy one-year-old lignified grapevine cuttings (approximately 50 cm long) from cvs. Cabernet Sauvignon, Sauvignon Blanc, and Malbec, were obtained from the Concha y Toro Vineyard, Research and Innovation Center, Fundo Pocoa, Pencahue (35°03′48″ S, 71°16′19″ W), Maule Region, Chile. Cuttings were transported to the Plant Pathology Laboratory at the University of Talca for surface disinfection. They were immersed in a 0.5% chlorine solution, rinsed with sterile water, and air-dried on absorbent paper at room temperature (20–22 °C). For each combination of isolate and pruning wound age, three replicate experimental units were established for each cultivar. Each experimental unit consisted of 15 cuttings, each bearing a single pruning wound, resulting in a total of 45 cuttings per treatment. Cuttings were vertically placed at a 90° angle and buried 10 cm deep in disinfected polyethylene containers (35 × 30 × 15 cm) filled with sterile moist perlite. Pruning was performed at the apex using sterilized pruning shears, making a 45° bevel cut. At each pruning wound age (1, 15, 30, 45, and 60 days after pruning), a separate set of cuttings was inoculated with 40 µL of a mycelial suspension (10^5^ mycelial fragments/mL) directly applied onto the wound surface. Control cuttings received 40 µL of sterile distilled water. All cuttings were maintained for five months in a greenhouse located in Talca (35°24′ S, 71°37′ W), with an average temperature of 15.7 °C and 69.0% relative humidity (RH). Lesion lengths were measured using a digital caliper, from the wound margin to the distal edge of the necrotic area. Data from both repetitions of the experiment were pooled for analysis.

To determine wound infection (%), five wood fragments (approximately 5 mm in diameter) were excised from the margins of necrotic lesions at each inoculation site. Fragments were surface disinfected with 75% ethanol, air-dried under sterile conditions in a laminar flow hood, and plated onto 2% potato dextrose agar (PDA) [28]. Plates were incubated at 25 °C under a 12 h light/dark photoperiod. Fungal re-isolation was confirmed based on colony morphology, growth rate, and microscopic examination of conidia. Wound infection (%) was calculated as the proportion of pruning wounds from which the inoculated fungus was successfully re-isolated [19,28].

### 2.3. Susceptibility of Grapevine Pruning Wounds to Botryosphaeriaceae Infection Under Vineyard Conditions

Field studies were conducted at the El Llano vineyard using cvs. Cabernet Sauvignon and Merlot, which were trained as bilateral cordons under a drip irrigation system, located in San Clemente (35°33′ S, 71°28′ W), Maule Region, Central Chile.

The experiment was arranged as a randomized complete block design with three replicates. For each combination of isolate and pruning wound age, one experimental unit was established per replicate for each cultivar. Each experimental unit consisted of 15 pruning wounds (canes) distributed on independent grapevine plants. Experimental units were spatially separated and located on different plants and rows within the vineyard to account for field variability.

During the last week of July, canes were pruned at a 45° angle using disinfected pruning shears, leaving 3 to 4 buds per cane [17]. Pruning wounds were inoculated with 40 µL of a mycelial suspension (10^5^ mycelial fragments/mL) at 1, 15, 30, 45, and 60 d after pruning. Control wounds received 40 µL of sterile distilled water.

Canes were collected for laboratory analysis eight months after inoculation, and lesion length (mm) was measured using a digital caliper from the wound margin to the edge of the necrosis.

Wound infection (%) was determined as described above for greenhouse experiments. Briefly, five wood fragments (approximately 5 mm in diameter) were taken from the margins of necrotic lesions at each inoculation site, surface disinfected, and plated onto 2% PDA [28]. Plates were incubated at 25 °C under a 12 h light/dark photoperiod, and fungal re-isolation was confirmed based on colony morphology, growth rate, and microscopic observation of conidia [19,28].

### 2.4. Data Analysis

Data were analyzed separately for the greenhouse and vineyard experiments. For both experiments, fungal isolate and pruning wound age were included as fixed factors in the models. Data were analyzed separately for each cultivar to better characterize isolate-specific effects and differences in susceptibility over time. Lesion length (mm) and wound infection (%) were analyzed independently. Lesion length data were analyzed using a generalized linear model (GLM) with a Gamma distribution and log link function. Wound infection (%) was analyzed using a binomial GLM with a logit link function. When significant interactions between isolate and pruning wound age were detected, pairwise comparisons of estimated marginal means (EMMs) were performed using the *emmeans* package in R. Differences were considered significant at *p* < 0.05, with Sidak adjustment for multiple comparisons. All statistical analyses were performed using R version 4.1.0.

## 3. Results

### 3.1. Susceptibility of Grapevine Pruning Wounds to Botryosphaeriaceae Infection Under Greenhouse Conditions

All grapevine cuttings were viable and developed roots and shoots under greenhouse conditions. Inoculated cuttings of all cultivars developed visible necrotic lesions at all pruning wound ages.

The GLM analysis revealed highly significant effects of isolate, pruning wound age, and their interaction for each cultivar (*p* < 0.001 for all factors), indicating that lesion length was influenced by both factors and their interaction (Table 1).

For each cultivar, lesion length was highest at 1 day after pruning and decreased with increasing pruning wound age. This reduction was most pronounced between 1 and 15 d, followed by a more gradual decline until 60 d (Figure S1A–C).

For the cv. Cabernet Sauvignon, *N. parvum* from grapevine (Np-vine) and *N. arbuti* from apple (Na-apple) produced the largest lesions at early wound ages, whereas *D. seriata* from apple (Ds-apple) and *Do. sarmentorum* from walnut (Dsar-wal) were among the least aggressive isolates. Similar trends were observed for the cvs. Malbec and Sauvignon Blanc, with lesion length markedly reduced by 60 d after pruning for all isolates (Table 1). For example, in cv. Cabernet Sauvignon, mean lesion length declined from 211.8 mm at 1 day to 35.0 mm at 60 d for Np-vine. Control treatments consistently resulted in minimal lesion development across all pruning wound ages.

The GLM analysis of wound infection (%) also revealed highly significant effects of isolate, pruning wound age, and their interaction for each cultivar (*p* < 0.001 for all factors; Table 2).

For each cultivar, wound infection (%) was highest at 1 d after pruning and decreased with increasing pruning wound age (Figure S2A–C). Most isolates caused high wound infection (%) at early wound ages, followed by a marked reduction between 1 and 15 d and a continued decline through 60 d. For example, in cv. Cabernet Sauvignon, the overall mean wound infection decreased from 80.6% at 1 d to 18.1% at 60 d. Control treatments showed consistently low wound infection (%) throughout the experiment.

### 3.2. Susceptibility of Grapevine Pruning Wounds to Botryosphaeriaceae Infection Under Vineyard Conditions

The GLM analysis revealed highly significant effects of isolate, pruning wound age, and their interaction for each cultivar under vineyard conditions (*p* < 0.001 for all factors), indicating that lesion length was influenced by both factors and their interaction (Table 3).

For each cultivar, lesion length was highest at 1 d after pruning and decreased with increasing pruning wound age (Figure S1D,E). This reduction was less pronounced than that observed under greenhouse conditions but still showed a decreasing trend over time. The significant interaction between isolate and pruning wound age indicated that the magnitude of this reduction varied depending on the isolate.

For the cv. Cabernet Sauvignon, *N. parvum* from grapevine (Np-vine) and *N. arbuti* from apple (Na-apple) produced the largest lesions at early wound ages, whereas *Do. sarmentorum* from walnut (Dsar-wal) was among the least aggressive isolates. Similar trends were observed for the cv. Merlot, with lesion development decreasing over time for all isolates (Table 3). For example, in cv. Cabernet Sauvignon, the overall mean lesion length declined from 47.5 mm at 1 day to 25.1 mm at 60 d. Control treatments consistently resulted in minimal lesion development across all pruning wound ages.

The GLM analysis of wound infection (%) also revealed highly significant effects of isolate, pruning wound age, and their interaction for each cultivar (*p* < 0.001 for all factors; Table 4).

For each cultivar, wound infection (%) was highest at 1 d after pruning and decreased with increasing pruning wound age. Most isolates caused high wound infection (%) at early wound ages, followed by a marked reduction between 1 and 15 d and a continued decline through 60 d, consistent with the pattern observed for lesion length (Figure S2D,E). For example, in cv. Cabernet Sauvignon, the overall mean wound infection (%) decreased from 74.9% at 1 d to 15.5% at 60 d. Similar trends were observed for the cv. Merlot. Control treatments consistently showed low wound infection (%) throughout the experiment. No Botryosphaeriaceae species were recovered from non-inoculated control plants. Instead, fungi such as *Alternaria* sp., *Penicillium* sp., and *Epicoccum* sp. were occasionally isolated, consistent with their role as common saprophytes colonizing pruning wounds.

## 4. Discussion

The results obtained in the present study, including both greenhouse and field experiments, revealed a consistent pattern of decreasing pruning wound susceptibility with increasing wound age. Both lesion length and infection incidence declined progressively over time, confirming that pruning wounds are most vulnerable immediately after pruning and become less susceptible as wound healing progresses [1,17,19,20,29,30,31]. Importantly, our findings provide novel evidence supporting the epidemiological relevance of host jumping within Botryosphaeriaceae under field-relevant conditions. To our knowledge, this is the first study demonstrating that isolates originating from different fruit hosts can successfully infect grapevine pruning wounds, highlighting the potential for cross-host transmission in mixed agricultural landscapes of central Chile [23]. Most previous studies have been conducted using Botryosphaeriaceae obtained from the same host (grapevine) in the Northern Hemisphere [17,20,21,22,29], with comparatively fewer studies in the Southern Hemisphere [19,28,30,31]. In agreement with this body of literature, our findings indicate that pruning wounds can remain susceptible for up to 60 days after pruning, with the highest levels of infection occurring during the first two weeks, followed by a gradual decline over time [1,17,20,21,29,31].

An important contribution of this study is the evaluation of isolates obtained not only from grapevine but also from apple, blueberry, and walnut. This is epidemiologically relevant because vineyards in central Chile frequently coexist with other fruit crops that may serve as alternative inoculum reservoirs. Under both greenhouse and vineyard conditions, the isolates of *N. parvum* obtained from grapevine and *N. arbuti* obtained from apple were among the most aggressive, consistently producing the largest lesions. In contrast, isolates such as *D. seriata* from apple and *Do. sarmentorum* from walnut were less aggressive, although still pathogenic on grapevine pruning wounds. These results indicate that pathogenicity on grapevine was not restricted to isolates originally obtained from grapevine, but also included isolates recovered from other fruit hosts. This finding agrees with previous reports describing the broad host range of Botryosphaeriaceae species and their capacity for cross-infection among woody perennial crops [23,32,33]. In this context, the present study extends previous evidence by showing that isolates recovered from apple, blueberry, and walnut can infect grapevine pruning wounds over a wide range of wound ages. Therefore, the risk posed by Botryosphaeriaceae in viticultural systems should not be considered exclusively within-vineyard, but rather within a broader agricultural landscape where multiple fruit crops may contribute to inoculum pressure.

The temporal decline in pruning wound susceptibility observed in this study is consistent with previous findings in different viticultural regions. For instance, Díaz and Latorre [28] evaluated the susceptibility of grapevine pruning wounds to *Phaeomoniella chlamydospora* in Chile and reported a marked decrease in the percentage of infected wounds over time, with infection rates declining from approximately 97% on day 1 to less than 20% by day 45. Similarly, Sosnowski et al. [18], working in Australian vineyards with Botryosphaeriaceae species, found that susceptibility significantly declined after the first 14 days post-pruning. The present findings reinforce these observations, indicating that under both greenhouse and field conditions, the first 15 days after pruning represent a critical period of high vulnerability for pruning wounds.

This progressive decrease in pruning wound susceptibility has been associated with physiological healing processes influenced by environmental factors, particularly temperature [19,29,34]. In grapevines, pruning-induced tylosis has been reported to develop rapidly during the first week after wounding, although not all vessels become completely occluded [35]. In parallel, the suberization of wounded tissues, a key defense mechanism against pathogen entry, progresses more efficiently under higher temperatures [36]. In contrast, pruning in this study was performed during the Southern Hemisphere winter (July–August), when lower temperatures may have slowed wound healing processes and contributed to the extended susceptibility period observed up to 60 d.

Climatic conditions also play a key role in determining inoculum availability. Úrbez-Torres et al. [37] reported in California that 60% of Botryosphaeriaceae spores were captured following winter rainfall events, whereas spore release was markedly lower during autumn and early spring (22%) and nearly absent in late spring and summer (3%). Similarly, van Niekerk et al. [31,38] found in South Africa that periods of high humidity and precipitation coincided with the highest spore trap counts, which might help explain the greater infection risk in fresh pruning wounds. These reports are consistent with the conditions under which the present study was conducted, since pruning took place during the winter period in central Chile, when rainfall events were frequent. In addition, Valdez-Ténezaca et al. [39] showed in Chilean apple orchards that more than 70% of Botryosphaeriaceae inoculum was released during winter rainfall events between June and August. These findings supported the idea that high inoculum availability during the pruning period likely contributed to the elevated wound infection observed in young wounds and may also explain why susceptibility persisted for several weeks. Moreover, given the proximity between vineyards and other fruit crops in central Chile, these hosts may act as additional inoculum sources, facilitating cross-infection events [23,32,33].

The timing of pruning may represent a key management strategy to reduce the incidence of diseases caused by Botryosphaeriaceae species [1,34]. Several studies have evaluated the effects of early versus late pruning on pruning wound infection, with contrasting results depending on the region. In California, Úrbez-Torres and Gubler [29] demonstrated that wounds pruned in early winter (November–January) remained susceptible for up to 84 d, whereas those pruned in early spring (March) were only susceptible for 12 d. In contrast, studies conducted in Spain and South Africa reported greater susceptibility in wounds pruned toward the end of winter (February–March) compared with those pruned earlier [31,40]. In line with these findings, Rosace et al. [34] observed that although pruning wound susceptibility decreased over time regardless of pruning timing, the decline occurred more rapidly when pruning was performed later in the season. In the present study, conducted under early winter pruning conditions, high susceptibility was observed during the first two weeks after pruning, followed by a progressive reduction up to 45 d. However, low levels of infection were still detected at 60 d, indicating that susceptibility, although reduced, may persist for up to two months after pruning. Evaluating pruning at later dates under Chilean conditions would help determine how temperature and inoculum availability influence the duration of susceptibility.

Although identifying the period of greatest susceptibility of pruning wounds is crucial to reduce infection risk, this must be complemented with effective wound protection strategies. The application of fungicides or biocontrol agents immediately after pruning has been shown to reduce pathogen entry into woody tissues [1,19]. However, the duration of this protection remains uncertain, and pruning wounds in this study remained susceptible for up to 60 d, suggesting that a single application may be insufficient to provide sufficient protection throughout the susceptibility period. Accordingly, management based on a single fungicide application might be insufficient [18,41], and various applications may be required to maintain effective protection over time. These findings highlight the need to evaluate protection strategies aligned with the duration of wound susceptibility, including repeated applications and the combined use of fungicides and biocontrol agents.

Finally, this study provides the first evidence that Botryosphaeriaceae isolates from different fruit hosts can infect grapevine pruning wounds, supporting the epidemiological relevance of host jumping and highlighting alternative hosts as potential inoculum reservoirs in central Chile. However, some limitations of this study should be acknowledged. Future studies incorporating multiple seasons, natural inoculum sources, an increased number of isolates representing different host origins, and detailed climatic monitoring would improve our understanding of pruning wound susceptibility and its relationship with environmental conditions.

## Figures and Tables

**Table 1:** Mean lesion length (mm) on grapevine cuttings (cv. Cabernet Sauvignon, Malbec, and Sauvignon Blanc) inoculated with different Botryosphaeriaceae isolates and evaluated at five pruning wound ages (1, 15, 30, 45, and 60 d) under greenhouse conditions. Lesions were caused by artificial inoculation with 40 µL of a mycelial suspension (10^5^ mycelial fragments/mL) applied to pruning wounds of different ages over a five-month period.

Isolate Code ^y^	Mean Lesion Length (mm) ^x^					
	cv. Cabernet Sauvignon					
	1 Day	15 Days	30 Days	45 Days	60 Days	Mean
Ds-vine	29.6 fg	28.4 f–h	16.4 i-m	12.2 k–o	10.1 no	19.3
Np-vine	211.8 a	194.9 ab	119.0 c	37.3 ef	35.0 ef	119.6
Ds-apple	18.0 i–k	16.8 i–l	14.0 j–m	12.3 k–o	11.2 m–o	14.5
Dm-blue	55.9 d	33.3 e–g	12.4 k–o	10.6 no	9.4 no	24.3
Na-apple	177.4 ab	140.2 bc	49.3 de	37.0 ef	11.5 l–o	83.1
Dsar-wal	47.2 de	23.2 g–i	19.4 h–j	11.9 l–o	8.9 o	22.1
Control ^z^	5.5 p	4.0 p	4.5 p	4.1 p	3.9 p	4.4
Mean	77.9	62.9	33.6	18.0	12.9	
**GLM**		df	*Chi* ^2^	*p*		
Isolate (I)		6	509.77	<0.001		
Age (A)		4	130.43	<0.001		
I × A		24	56.16	<0.001		
**Isolate Code ^y^**	**cv.** **Malbec**					
	**1 Day**	**15 Days**	**30 Days**	**45 Days**	**60 Days**	**Mean**
Ds-vine	60.7 a–c	23.8 gh	22.8 gh	13.3 ij	8.9 j–m	25.9
Np-vine	89.2 a	78.0 ab	24.9 gh	17.4 hi	18.5 g–i	45.6
Ds-apple	63.5 a–c	59.0 b–c	11.4 j–l	8.8 k–m	7.5 mn	30.0
Dm-blue	88.2 a	38.1 ef	13.2 ij	12.6 i–k	8.4 lm	32.1
Na-apple	71.1 ab	46.5 c–e	39.8 de	26.4 fg	11.6 j–l	39.1
Dsar-wal	20.1 gh	20.8 gh	17.6 hi	9.6 j–m	9.2 j–m	15.5
Control ^z^	5.1 no	5.0 o	4.1 0	4.2 o	4.2 o	4.5
Mean	56.8	38.7	19.1	13.2	9.8	
**GLM**		df	*Chi* ^2^	*p*		
Isolate (I)		6	202.15	<0.001		
Age (A)		4	173.20	<0.001		
I × A		24	56.20	<0.001		
**Isolate Code ^y^**	**cv.** **Sauvignon Blanc**					
	**1 Day**	**15 Days**	**30 Days**	**45 Days**	**60 Days**	**Mean**
Ds-vine	32.1 f–h	21.9 h–j	14.0 k–m	14.3 j–m	8.6 n–p	18.2
Np-vine	126.6 ab	113.4 a–c	64.7 de	48.0 ef	9.4 n–p	72.4
Ds-apple	26.9 g–i	17.8 i–k	18.2 i–k	14.7 j–l	8.8 n–p	17.3
Dm-blue	73.6 c–e	36.9 fg	19.8 i–k	10.3 l–o	8.2 op	29.8
Na-apple	135.4 a	85.0 b–d	60.4 de	13.1 k–n	9.8 l–o	60.7
Dsar-wal	36.4 fg	32.3 f–h	27.2 g–i	10.2 l–o	7.3 o–q	22.7
Control ^z^	6.1 p–r	5.2 qr	4.2 r	4.3 r	4.1 r	4.4
Mean	59.9	44.8	27.6	15.7	7.7	
**GLM**		df	*Chi* ^2^	*p*		
Isolate (I)		6	314.32	<0.001		
Age (A)		4	155.61	<0.001		
I × A		24	60.87	<0.001		

^x^ Different lowercase letters within columns indicate statistically significant differences (*p* < 0.05) among isolate–wound age combinations within each cultivar; letter ranges include all intermediate letters. ^y^ Isolates code Isolate codes correspond to species and host of origin: Np-vine (*Neofusicoccum parvum* from grapevine), Na-apple (*Neofusicoccum arbuti* from apple), Dm-blue (*Diplodia mutila* from blueberry), Dsar-wal (*Dothiorella sarmentorum* from walnut), Ds-vine (*Diplodia seriata* from grapevine), Ds-apple (*Diplodia seriata* from apple), and the non-inoculated control. ^z^ Negative control treatment inoculated using 40 µL of sterile distilled water.

**Table 2:** Wound infection (%) on grapevine cuttings (cv. Cabernet Sauvignon, Malbec, and Sauvignon Blanc) inoculated with different Botryosphaeriaceae isolates and evaluated at five pruning wound ages (1, 15, 30, 45, and 60 d) under greenhouse conditions. Lesions were caused by artificial inoculation with 40 µL of a mycelial suspension (10^5^ mycelial fragments/mL) applied to pruning wounds at different ages over a five-month period.

Isolate Code ^y^	Wound Infection (%) ^x^					
	cv. Cabernet Sauvignon					
	1 Day	15 Days	30 Days	45 Days	60 Days	Mean
Ds-vine	99.3 a	80.7 bc	59.3 d–i	41.3 i–l	21.3 m–p	60.4
Np-vine	97.3 a	82.0 bc	58.7 d–j	52.7 e–k	28.0 l–o	63.7
Ds-apple	90.7 ab	67.3 c–g	51.3 f–k	34.0 k–n	18.7 n–r	52.4
Dm-blue	92.0 ab	70.7 c–e	46.7 i–k	40.0 j–l	14.0 o–s	52.7
Na-apple	93.3 ab	71.3 cd	68.7 c–f	47.3 h–k	20.7 m–q	60.3
Dsar-wal	80.7 bc	66.0 c–h	49.3 g–k	36.0 k–m	18.7 n–r	50.1
Control ^z^	7.3 q–s	8.0 p–s	7.3 q–s	6.0 rs	5.3 s	6.8
Mean	80.6	63.7	48.8	36.8	18.1	
**GLM**		df	*Chi* ^2^	*p*		
Isolate (I)		6	79.20	<0.001		
Age (A)		4	121.07	<0.001		
I × A		24	10.37	<0.001		
**Isolate Code ^y^**	**cv.** **Malbec**					
	**1 Day**	**15 Days**	**30 Days**	**45 Days**	**60 Days**	**Mean**
Ds-vine	90.7 ab	62.0 f–h	32.7 k–o	28.7 l–p	10.7 r–v	45.0
Np-vine	98.0 a	80.0 b–e	60.0 f–i	46.0 h–l	12.0 q–v	59.2
Ds-apple	70.0 d–g	66.0 e–g	52.7 g–j	26.0 m–q	14.7 p–v	45.9
Dm-blue	84.0 b–d	55.3 g–j	24.7 n–r	21.3 o–u	9.3 t–v	38.9
Na-apple	88.0 a–c	73.3 c–f	39.3 j–n	43.3 i–m	16.7 p–v	52.1
Dsar-wal	82.7 b–d	46.7 h–k	24.0 n–s	22.0 o–t	10.0 s–v	37.1
Control ^z^	8.0 uv	7.3 v	6.7 v	8.7 t–v	8.0 uv	7.7
Mean	74.5	55.8	34.3	28.0	11.6	
**GLM**		df	*Chi* ^2^	*p*		
Isolate (I)		6	58.15	<0.001		
Age (A)		4	126.21	<0.001		
I × A		24	16.05	<0.001		
**Isolate Code ^y^**	**cv.** **Sauvignon Blanc**					
	**1 Day**	**15 Days**	**30 Days**	**45 Days**	**60 Days**	**Mean**
Ds-vine	80.7 b–e	78.0 c–f	46.7 ij	25.3 kl	12.7 l–q	48.7
Np-vine	88.7 a–c	90.7 ab	60.0 g–i	46.0 ij	26.7 k	62.4
Ds-apple	88.0 a–c	68.0 e–h	56.0 hi	20.0 k–o	18.0 k–p	50.0
Dm-blue	84.7 a–d	73.3 d–g	55.3 hi	24.0 k–m	12.0 m–q	49.9
Na-apple	92.7 a	79.3 b–f	66.0 f–h	31.3 jk	21.3 k–n	58.1
Dsar-wal	79.3 b–f	66.7 e–h	48.0 i	23.3 k–n	11.3 n–q	45.7
Control ^z^	8.0 pq	9.3 o–q	6.7 q	6.7 q	5.3 q	7.2
Mean	74.4	66.5	48.4	25.2	15.3	
**GLM**		df	*Chi* ^2^	*p*		
Isolate (I)		6	69.03	<0.001		
Age (A)		4	134.42	<0.001		
I × A		24	7.91	<0.001		

^x^ Different lowercase letters within columns indicate statistically significant differences (*p* < 0.05) among isolate–wound age combinations within each cultivar; letter ranges include all intermediate letters. ^y^ Isolates code Isolate codes correspond to species and host of origin: Np-vine (*Neofusicoccum parvum* from grapevine), Na-apple (*Neofusicoccum arbuti* from apple), Dm-blue (*Diplodia mutila* from blueberry), Dsar-wal (*Dothiorella sarmentorum* from walnut), Ds-vine (*Diplodia seriata* from grapevine), Ds-apple (*Diplodia seriata* from apple), and the non-inoculated control. ^z^ Negative control treatment inoculated using 40 µL of sterile distilled water.

**Table 3:** Mean lesion length (mm) on canes (cv. Cabernet Sauvignon and Merlot) inoculated with different Botryosphaeriaceae isolates and evaluated at five pruning wound ages (1, 15, 30, 45, and 60 d) under vineyard conditions. Lesions were caused by artificial inoculation with 40 µL of a mycelial suspension (10^5^ mycelial fragments/mL) applied to pruning wounds at different ages over an eight-month period.

Isolate Code ^y^	Mean Lesion Length (mm) ^x^					
	cv. Cabernet Sauvignon					
	1 Day	15 Days	30 Days	45 Days	60 Days	Mean
Ds-vine	52.2 b–e	48.1 b–g	39.9 f–i	38.3 g–i	33.1 h–j	42.3
Np-vine	69.1 a	56.2 a–c	47.5 b–g	40.6 e–i	38.3 g–i	50.3
Ds-apple	49.9 b–f	31.9 ij	38.7 g–i	28.6 j	21.0 k	34.0
Dm-blue	53.8 a–d	46.5 c–g	44.8 c–g	40.0 f–i	28.0 j	42.6
Na-apple	60.6 ab	57.6 a–c	53.8 a–d	52.2 b–e	38.8 f–i	52.6
Dsar-wal	42.5 d–h	27.1 j	25.7 jk	20.7 k	12.8 l	25.8
Control ^z^	4.5 m	4.9 m	4.2 m	4.4 m	4.0 m	4.4
Mean	47.5	38.9	36.4	32.1	25.1	
**GLM**		df	*Chi* ^2^	*p*		
Isolate (I)		6	218.52	<0.001		
Age (A)		4	21.26	<0.001		
I × A		24	7.06	<0.001		
**Isolate Code ^y^**	**cv.** **Merlot**					
	**1 Day**	**15 Days**	**30 Days**	**45 Days**	**60 Days**	**Mean**
Ds-vine	42.8 a–c	40.2 a–d	34.5 b–f	21.5 hi	14.3 j–l	30.7
Np-vine	47.8 a	42.9 a–c	35.5 b–f	29.3 e–g	21.8 hi	35.5
Ds-apple	40.3 a–d	40.7 a–d	33.8 b–f	23.8 g–i	14.9 jk	30.7
Dm-blue	39.5 a–d	31.0 d–g	28.7 f–h	18.4 ij	12.8 kl	24.7
Na-apple	48.3 a	44.5 ab	39.2 a–e	31.0 d–g	17.8 ij	36.2
Dsar-wal	36.1 a–f	32.1 c–f	14.4 j–l	12.7 kl	10.8 l	21.2
Control ^z^	5.4 m	2.5 o	2.8 no	3.7 n	3.3 no	3.5
Mean	41.5	33.4	27.0	20.0	12.9	
**GLM**		df	*Chi* ^2^	*p*		
Isolate (I)		6	194.24	<0.001		
Age (A)		4	54.59	<0.001		
I × A		24	16.96	<0.001		

^x^ Different lowercase letters within columns indicate statistically significant differences (*p* < 0.05) among isolate–wound age combinations within each cultivar; letter ranges include all intermediate letters. ^y^ Isolates code Isolate codes correspond to species and host of origin: Np-vine (*Neofusicoccum parvum* from grapevine), Na-apple (*Neofusicoccum arbuti* from apple), Dm-blue (*Diplodia mutila* from blueberry), Dsar-wal (*Dothiorella sarmentorum* from walnut), Ds-vine (*Diplodia seriata* from grapevine), Ds-apple (*Diplodia seriata* from apple), and the non-inoculated control. ^z^ Negative control treatment inoculated using 40 µL of sterile distilled water.

**Table 4:** Wound infection (%) on canes (cv. Cabernet Sauvignon and Merlot) inoculated with different Botryosphaeriaceae isolates and evaluated at five pruning wound ages (1, 15, 30, 45, and 60 d) under vineyard conditions. Lesions were caused by artificial inoculation with 40 µL of a mycelial suspension (10^5^ mycelial fragments/mL) applied to pruning wounds at different ages over an eight-month period.

Isolate Code ^y^	Wound Infection (%) ^x^					
	cv. Cabernet Sauvignon					
	1 Day	15 Days	30 Days	45 Days	60 Days	Mean
Ds-vine	85.3 a–c	70.0 d–f	32.7 j–n	42.0 i–k	12.7 pq	48.5
Np-vine	91.3 a	74.7 b–e	53.3 f–i	32.7 j–n	22.7 l–q	54.9
Ds-apple	80.7 a–d	70.7 c–f	47.3 g–j	28.0 k–o	16.0 o–q	48.5
Dm-blue	87.3 ab	60.0 e–h	42.7 h–k	24.0 l–p	18.7 n–q	46.5
Na-apple	87.3 ab	74.0 b–e	44.0 h–k	36.0 i–m	19.3 n–q	52.1
Dsar-wal	80.0 a–d	63.3 e–g	39.3 i–l	20.7 m–q	16.0 o–q	43.9
Control ^z^	12.7 pq	12.0 pq	11.3 pq	9.3 q	9.3 q	10.9
Mean	74.9	60.7	38.7	27.5	15.5	
**GLM**		df	*Chi* ^2^	*p*		
Isolate (I)		6	46.16	<0.001		
Age (A)		4	113.42	<0.001		
I × A		24	10.45	<0.001		
**Isolate Code ^y^**	**cv.** **Merlot**					
	**1 Day**	**15 Days**	**30 Days**	**45 Days**	**60 Days**	**Mean**
Ds-vine	83.3 ab	78.0 a–c	42.0 fg	30.0 g–j	14.7 jk	49.6
Np-vine	88.7 a	83.3 ab	50.7 ef	53.3 d–f	19.3 i–k	59.1
Ds-apple	70.7 b–d	57.3 d–f	38.7 f–h	31.3 g–i	16.0 i–k	42.8
Dm-blue	86.7 a	62.0 c–e	38.7 f–h	19.3 i–k	16.7 i–k	44.7
Na-apple	82.7 ab	79.3 ab	45.3 e–g	29.3 g–j	16.7 i–k	50.7
Dsar-wal	78.7 a–c	70.0 b–d	27.3 g–j	21.3 h–k	10.7 k	41.6
Control ^z^	10.0 k	14.7 jk	9.3 k	11.3 k	8.0 k	10.7
Mean	71.0	63.5	36.0	28.0	14.6	
**GLM**		df	*Chi* ^2^	*p*		
Isolate (I)		6	48.92	<0.001		
Age (A)		4	115.52	<0.001		
I × A		24	11.61	<0.001		

^x^ Different lowercase letters within columns indicate statistically significant differences (*p* < 0.05) among isolate–wound age combinations within each cultivar; letter ranges include all intermediate letters. ^y^ Isolates code Isolate codes correspond to species and host of origin: Np-vine (*Neofusicoccum parvum* from grapevine), Na-apple (*Neofusicoccum arbuti* from apple), Dm-blue (*Diplodia mutila* from blueberry), Dsar-wal (*Dothiorella sarmentorum* from walnut), Ds-vine (*Diplodia seriata* from grapevine), Ds-apple (*Diplodia seriata* from apple), and the non-inoculated control. ^z^ Negative control treatment inoculated using 40 µL of sterile distilled water.

## Data Availability

The datasets used and/or analyzed during the current study are available from the corresponding author on request.

## Supplementary Materials

The following supporting information can be downloaded at https://www.mdpi.com/article/10.3390/jof12060424/s1: Figure S1: Lesion length (mm) in pruning wounds of grapevine cultivars inoculated with different Botryosphaeriaceae isolates under greenhouse (A–C) and vineyard (D,E) conditions. Lesion length was evaluated at 1, 15, 30, 45, and 60 days after pruning. Panels represent Cabernet Sauvignon (A,D), Malbec (B), Sauvignon Blanc (C), and Merlot (E). Each point represents the mean lesion length, and error bars indicate standard deviation. Isolates are identified by species and host of origin: Np-vine (*Neofusicoccum parvum* from grapevine), Na-apple (*Neofusicoccum arbuti* from apple), Dm-blue (*Diplodia mutila* from blueberry), Dsar-wal (*Dothiorella sarmentorum* from walnut), Ds-vine (*Diplodia seriata* from grapevine), Ds-apple (*Diplodia seriata* from apple), and a non-inoculated control; Figure S2: Wound infection (%) in pruning wounds of grapevine cultivars inoculated with different Botryosphaeriaceae isolates under greenhouse (A–C) and vineyard (D,E) conditions. Infection was evaluated at 1, 15, 30, 45, and 60 days after pruning. Panels represent Cabernet Sauvignon (A,D), Malbec (B), Sauvignon Blanc (C), and Merlot (E). Each point represents the mean infection, and error bars indicate standard deviation. Isolates are identified by species and host of origin: Np-vine (*Neofusicoccum parvum* from grapevine), Na-apple (*Neofusicoccum arbuti* from apple), Dm-blue (*Diplodia mutila* from blueberry), Dsar-wal (*Dothiorella sarmentorum* from walnut), Ds-vine (*Diplodia seriata* from grapevine), Ds-apple (*Diplodia seriata* from apple), and a non-inoculated control.
