# Supplementary material for: Temporal Susceptibility of Grapevine Pruning Wounds to Botryosphaeriaceae Host-Jumping Pathogens in Central Chile
Source: J Fungi (Basel). 2026 Jun 11;12(6):424. doi: 10.3390/jof12060424 (PMC13302271; doi:10.3390/jof12060424)
Supplement: Supplementary file 1 [file jof-12-00424-s001.zip › jof-4278227-supplementary.pdf]

Supplementary Material

Temporal Susceptibility of Grapevine Pruning Wounds to Botryosphaeriaceae Host-Jumping Pathogens in Central Chile

Yadira Hernández 1, Fernanda B. Núñez 1, Yuramis Quesada 1, Mauricio Lolas 1 and Karina Elfar 2, Akif Eskalen 2, Felipe Gainza-Cortés 3, Pedro E. Gundel 4,5, Eugenio Sanfuentes 6 and Gonzalo A. Díaz 1, *

1 Laboratory of Fruit Pathology, Faculty of Agricultural Sciences, University of Talca, Talca 3460000, Chile; yadira.hernandez@utalca.cl (Y.H.); fnunez@utalca.cl (F.B.N.); yuramis.quesada@utalca.cl (Y.Q.); mlolas@utalca.cl (M.L.)
2 Department of Plant Pathology, University of California, Davis, CA 95616, USA; kdelfar@ucdavis.edu (K.E.); eskalen@ucdavis.edu (A.E.)
3 Viña Concha y Toro S.A., Center for Research and Innovation, Fundo Pocoa s/n, Km10 Ruta K-650, Pencahue 3550000, Chile; felipe.gainza@conchaytoro.cl
4 Centro de Ecología Integrativa, Instituto de Ciencias Biológicas, Universidad de Talca, Campus Talca, Av. Lircay s/n, Talca 3460000, Chile; pedro.gundel@utalca.cl
5 Instituto de Biología Funcional y Biotecnología (BIOLAB)-INBIOTEC-CONICET-CICBA, Facultad de Agronomía, Universidad Nacional del Centro de la Provincia de Buenos Aires (UNICEN), Av. República de Italia # 780, Azul 7300, Buenos Aires, Argentina
6 Facultad de Ciencias Forestales y Centro de Biotecnología, Universidad de Concepción, Edmundo Larenas, 219, Concepción 4070409, Chile; esanfuen@udec.cl
* Correspondence: g.diaz@utalca.cl

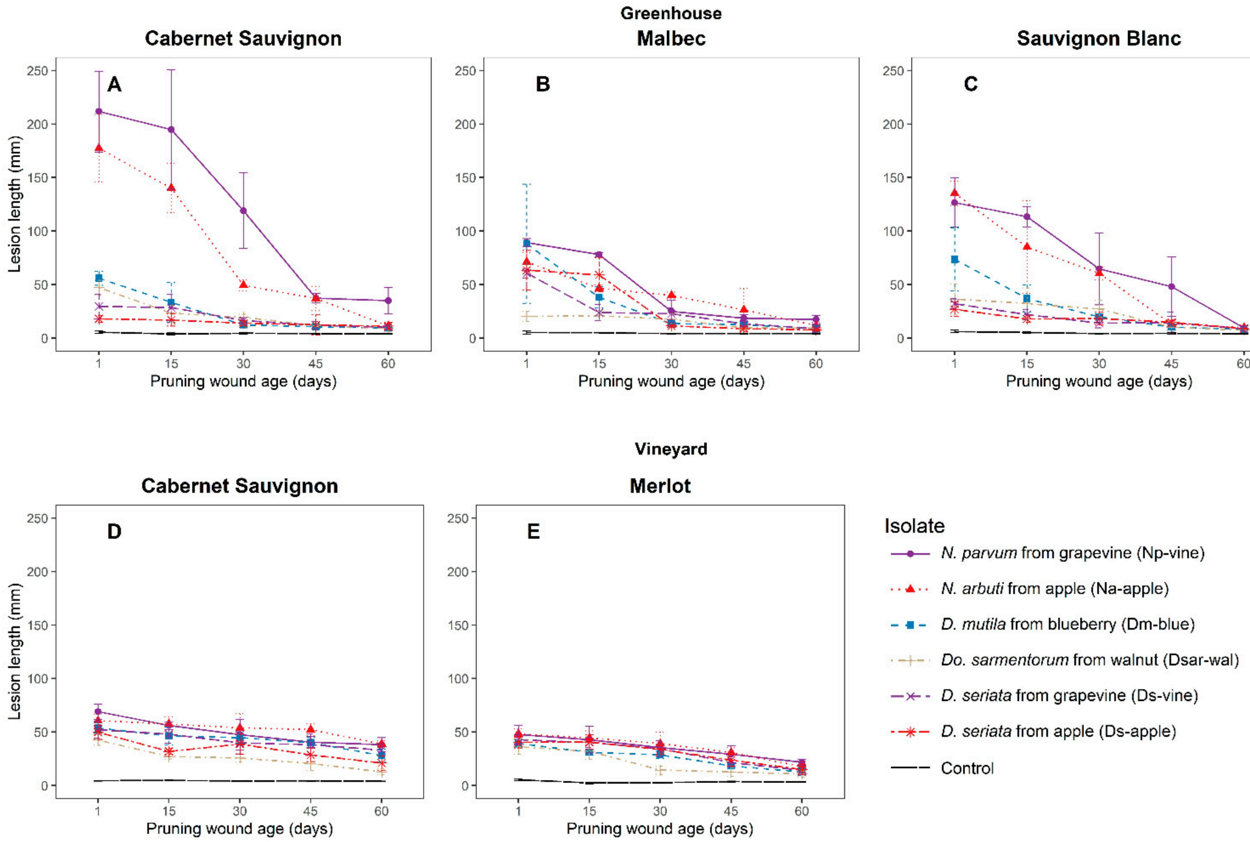

Figure S1. Lesion length (mm) in pruning wounds of grapevine cultivars inoculated with different Botryosphaeriaceae isolates under greenhouse (A–C) and vineyard (D–E) conditions. Lesion length was evaluated at 1, 15, 30, 45, and 60 days after pruning. Panels represent Cabernet Sauvignon (A, D), Malbec (B), Sauvignon Blanc (C), and Merlot (E). Each point represents the mean lesion length, and error bars indicate standard deviation. Isolates are identified by species and host of origin: Np-vine (*Neofusicoccum parvum* from grapevine), Na-apple (*Neofusicoccum arbuti* from apple), Dm-blue (*Diplodia mutila* from blueberry), Dsar-wal (*Dothiorella sarmentorum* from walnut), Ds-vine (*Diplodia seriata* from grapevine), Ds-apple (*Diplodia seriata* from apple), and a non-inoculated control.

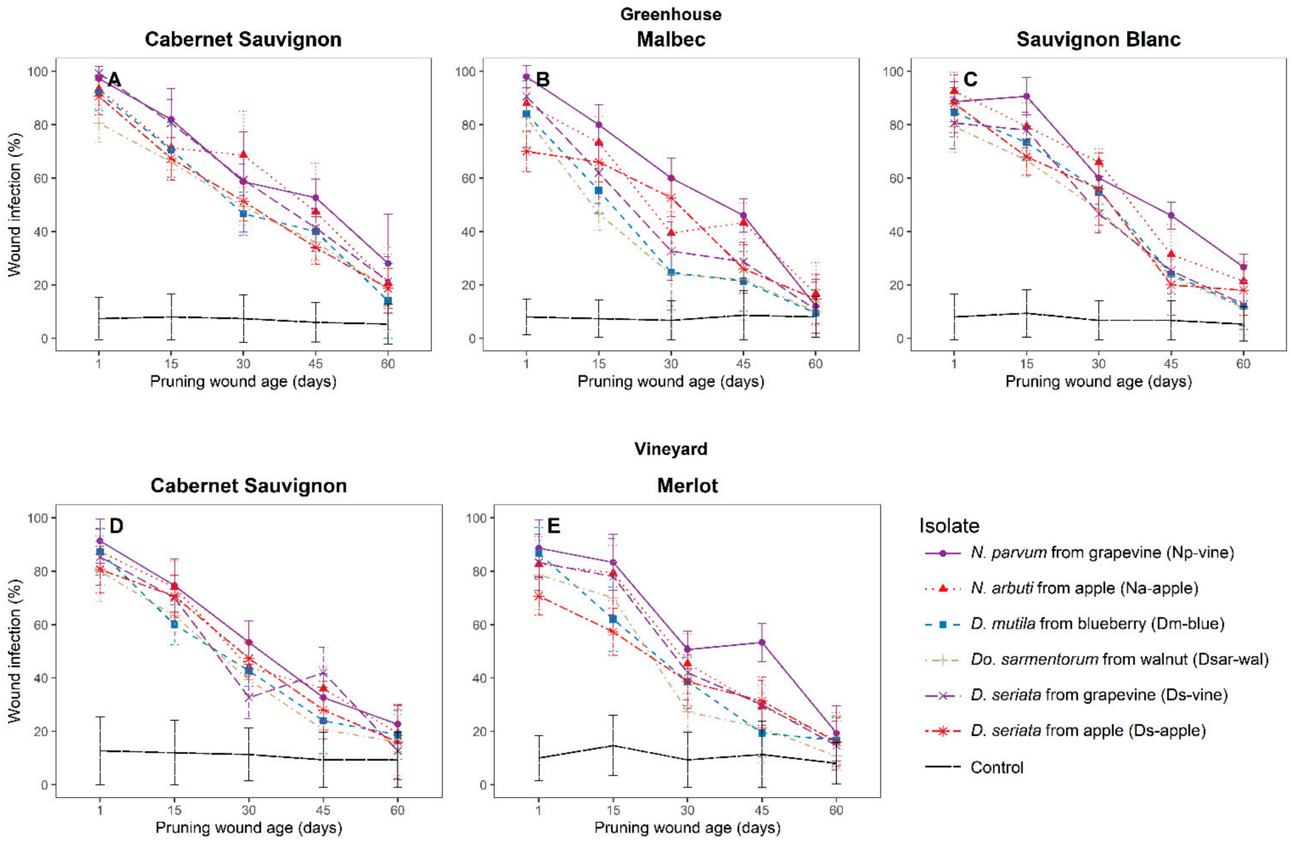

Figure S2. Wound infection (%) in pruning wounds of grapevine cultivars inoculated with different Botryosphaeriaceae isolates under greenhouse (A–C) and vineyard (D–E) conditions. Infection was evaluated at 1, 15, 30, 45, and 60 days after pruning. Panels represent Cabernet Sauvignon (A, D), Malbec (B), Sauvignon Blanc (C), and Merlot (E). Each point represents the mean infection, and error bars indicate standard deviation. Isolates are identified by species and host of origin: Np-vine (*Neofusicoccum parvum* from grapevine), Na-apple (*Neofusicoccum arbuti* from apple), Dm-blue (*Diplodia mutila* from blueberry), Dsar-wal (*Dothiorella sarmentorum* from walnut), Ds-vine (*Diplodia seriata* from grapevine), Ds-apple (*Diplodia seriata* from apple), and a non-inoculated control.
